# Reference Gene U2 Enables Direct Comparison between Relative Gene Expression Levels of Vascular Smooth Muscle Cells in Tissue and Culture Using Real-Time Quantitative PCR

**DOI:** 10.3390/cells12172135

**Published:** 2023-08-23

**Authors:** Christine Rager, Tobias Klöpper, Uwe Pfeil, Sabine Tasch, Michael Raymond Whittaker, Betty Exintaris, Andrea Mietens, Ralf Middendorff

**Affiliations:** 1 Signalling Transduction Group, Institute of Anatomy and Cell Biology, Faculty of Medicine, Justus-Liebig-University, 35392 Giessen, Germany; christine.rager@monash.edu (C.R.);; 2 Drug Discovery Disposition and Dynamics, Monash Institute of Pharmaceutical Sciences, Faculty of Pharmacy and Pharmaceutical Sciences, Monash University, Parkville, Melbourne, VIC 3052, Australia; 3 Cardiopulmonary Neurobiology Group, Institute of Anatomy and Cell Biology, Faculty of Medicine, Justus-Liebig-University, 35392 Giessen, Germany; 4 Drug Discovery Biology, Monash Institute of Pharmaceutical Sciences, Faculty of Pharmacy and Pharmaceutical Sciences, Monash University, Parkville, Melbourne, VIC 3052, Australia

**Keywords:** vascular smooth muscle cells, qPCR, reference gene (housekeeping gene), aorta

## Abstract

In nearly every lab, real-time quantitative polymerase chain reaction (qPCR) is used to quantify gene expression. However, a comparison of different samples requires the careful selection of suitable reference genes (RGs), sometimes referred to as housekeeping genes. In the case of vascular smooth muscle cells (vSMCs), it is important to know under which conditions gene expression in isolated and cultured vSMCs can be compared with vSMCs in a healthy blood vessel. We isolated the vSMC-containing layer of the rat aorta (tunica media) and used one (longitudinal) half for direct RNA extraction, while the other half served to isolate and culture vSMCs prior to RNA extraction. First, the expression of the routinely used RGs beta-actin (Actb) and Glyceraldehyde-3-phosphate dehydrogenase (Gapdh) is investigated in intact media and corresponding cultured vSMCs. Significant differences in their Ct values show that these RGs could not be used for such direct comparisons; therefore, we select 15 different RGs. Only the gene expression of the small ribonuclear protein (snRNP) U2 shows no significant differences between the absolute Ct values of cultured vSMCs and the intact media; moreover, no differences were found between male and female rats in our experimental setup. In conclusion, U2 was shown to be an appropriate (sex-independent) RG to compare relative expression levels of vSMCs in culture to those vSMCs within their physiological tissue environment.

## 1. Introduction

### 1.1. Relevance and Aim of This Study

In view of the extensive use of cell culture models, the issue of to what extent cultured cells actually reflect cells within their physiological environment in the tissues remains largely unsolved [1]. VSMCs are no exception, and thus, a direct comparison of vSMCs within intact blood vessels to their equivalents in vitro is intricate [2,3].

A transcription analysis may provide the most comprehensive view of changes due to cell isolation and culturing, both in a spatial and temporal resolution [4]. Thus, we used real-time qPCR to monitor differences in the gene expression in cultured vSMCs versus the intact media of rat aorta [5].

There are two ways to quantify the mRNA level for a gene of interest (GOI) measured by qPCR [6]. Besides absolute quantification, which requires serial dilutions of a cDNA sample and extensive calculations for a calibration curve [7], relative quantification by normalisation to an RG is preferred by most researchers [8]. With this method, the quality of qPCR not only depends on the chosen primers [9] and reaction conditions, but ultimately relies on the selection of an appropriate RG for normalisation [10].

Our aim was to determine an RG that is equally expressed in vSMCs within the intact aortic media and in the corresponding cells in culture, thus enabling their direct comparison at the level of gene expression. In addition, we provide an exemplary strategy to identify an RG for qPCR.

### 1.2. Quantitative PCR

Since its introduction by Mullis and colleagues in 1983 [11], polymerase chain reaction (PCR) has been under constant development. PCR is valued for its reliability and sensitivity [8] and thus has evolved into a standard technique used in scientific laboratories around the globe.

In general, PCR relies on the logarithmic amplification of a genetic sequence that is defined by the applied template sequence and the respective primers binding to it. PCR comprises three consecutive steps (denaturation, primer hybridisation, and elongation), each carried out at a specific temperature. The product can be detected in a qualitative manner according to its molecular size after electrophoresis [12]. In contrast, qPCR allows for the quantification of the amplification of a sample while following the reaction in real-time. In fluorescence-based qPCR, this is realised by the application of fluorescent reporter molecules [7,13].

The key value necessary for quantification is the cycle threshold (Ct). The Ct, also known as the quantification cycle (Cq) or crossing point (CP), is defined as the number of PCR cycles necessary to reach a defined fluorescence level or to exceed a fluorescence threshold level above the background level [13]. Assuming 100% efficiency of the PCR reaction, the original amount of target copies, along with the fluorescent signal intensity, is duplicated in each PCR cycle. The higher the GOI is expressed, the faster the cycle threshold is reached, thus yielding a smaller Ct value.

### 1.3. RGs and Their Requirements for Relative Gene Expression Quantification

The major advantage of relative gene expression quantification is the correction of variations in the levels of the detected gene expression of different samples. The variations are usually due to tissue or matrix effects, inconsistent RNA harvest [14], or cDNA synthesis by the enzymatic reaction of the reverse transcriptase (RT) [13].

A reliable quantification requires specific properties of the RG, which is an internal control gene with a sequence different to the GOI. First of all, a constant expression regardless of any experimental conditions. Furthermore, the RG should ideally be expressed within a similar expression range as the GOI [10]. In addition, it is important that the variance of the RG expression does not exceed the variance of the expression of the GOIs one would like to compare.

Housekeeping genes are thought to be essential for the maintenance of basic cell functions and are expressed at a constant level; therefore, they are often considered promising RG candidates [10]. Routinely used examples are ubiquitin, ribosomal or histone subunits, Actb, and Gapdh [15].

### 1.4. Aortic vSMCs and Cell Culture

The aorta, being the largest blood vessel in the body, is usually the main source for the extraction and investigation of cultured vSMCs [16,17]. The aorta is characterised by a thick tunica media, which is mainly composed of vSMCs and extracellular elastic fibres.

Within the intact media, vSMCs are spindle-shaped and contain a cigar-like central nucleus. The vSMCs are oriented circumferentially around the lumen, able to contract, and thus fulfil their major role in the regulation of blood pressure and blood flow by the adaption of the vessel’s diameter [18]. Within their healthy physiological environment and in association with neighbouring cells, vSMCs are characterised by a high degree of differentiation and a low level of proliferation [19]. Nevertheless, vSMCs adapt quickly upon exposure to changing environmental factors illustrating their high plasticity [19,20,21].

The singularisation of vSMCs for cell culture requires the destruction of the original vessel by enzymatic digestion and subsequent straining of the cells [22].

Once cells become detached from their physiological environment, their survival depends on a quick adaptation to the circumstances in vitro. In the case of vSMCs, this includes the loss of physiological contact and exchange with neighbouring cells [1,3,23,24], the lack of a constant supply of essential oxygen and nutrients through the bloodstream, the missing exposure to oscillating pressure changes (systole and diastole), and hence, the necessity to constantly adapt their cellular tone. In a nutshell, extracting cells from their original tissue and bringing them into culture has a tremendous impact and could rather mimic pathological conditions, such as blood vessel injury and disease, rather than a healthy tissue environment [25,26,27,28].

Finding an RG expressed stably enough throughout the entire experimental procedure from the intact media to cultured singularised vSMCs thus literally constitutes the search for a needle in the haystack.

## 2. Materials and Methods

### 2.1. Animals

Aortae were obtained from male and female Wistar rats (*Rattus norvegicus*) aged from 2.5 to 3 months and housed in the animal facility of the Veterinary Faculty at Justus-Liebig-University (JLU) Giessen, Germany. Housing, animal care, and related procedures were conducted according to the guidelines of the German Animal Welfare Act and approved by the Committee for Laboratory Animals of the JLU Giessen, case number JLU Nr. 577_M. Rats were anaesthetized with CO_2_ and sacrificed by cervical dislocation.

### 2.2. Tissue Preparation

Aortae were dissected from the heart to the bifurcation and immediately transferred into MEM 1× buffer (Gibco, Thermo Fisher Scientific, Waltham, MA, USA) with 1% (*v*/*v*) penicillin-streptomycin (Pen/Strep, 10,000 U/mL, Sigma Aldrich, St. Louis, MO, USA) and stored on ice. Preparation of the vSMC layer (tunica media) was performed in cold MEM 1× + Pen/Strep under a binocular microscope. The surrounding fat was removed using sterile tweezers, branching vessels were cut, and the aorta was bisected longitudinally. One-half of the same aorta was used for cell extraction (see chapter cell extraction), and the second half was prepared as follows.

The outer connective tissue layer (tunica adventitia) was removed manually with curved tweezers starting from the proximal end. The inner endothelial layer (tunica intima) was carefully stripped away. For RNA isolation, the moisture of the tissue was dabbed off, tissue was scaled and immediately frozen in liquid nitrogen until RNA isolation.

### 2.3. Histology-Paraffin Sectioning and Azan Staining

Rat tissue samples were prepared and fixed in Bouin’s solution for 24 h. Before embedding in paraffin, the tissue was dehydrated in an ascending ethanol series (70%, 80%, 90%, 100% (*v*/*v*)) and finally kept in xylol. Tissue was equilibrated in liquid paraffin at 65 °C overnight before the paraffin was left at room temperature to cool down. Tissues were cut into 5 µm thick sections with a microtome. Sections were dried at 40 °C overnight before staining.

After deparaffinisation and rehydration in a descending ethanol series and sterile water the tissue sections were stained according to Heidenhain’s protocol for Azan staining.

### 2.4. Immunofluorescence (IF) Staining of Paraffin-Embedded Tissue Sections

Deparaffinised and rehydrated tissue sections were incubated for one hour at room temperature in 2% (*v*/*v*) normal goat serum (Cat.no. G9023, Roche, Basel, Switzerland) in phosphate-buffered saline solution (PBS, at pH 7.4) to prevent non-specific binding of the antibodies. Indirect immunostaining was performed by a two-step approach.

Primary antibodies (Table 1) were adjusted to the respective concentrations in dilution buffer (PBS with 0.2% (*v*/*v*) BSA and 0.1% (*w*/*v*) NaN_3_), and tissue sections were incubated at 4 °C overnight. For negative controls, the primary antibodies were omitted.

Following two washing steps with PBS (10 min each), the sections were incubated with the secondary antibodies (Table 2) diluted to the respective concentrations in PBS for one hour at room temperature in darkness.

Nuclear staining was performed using DAPI (Cat.no. 10236276001, Roche, Basel, Switzerland) at a dilution of 1:1250 in PBS together with the secondary antibodies. Stained tissue sections were rinsed twice with PBS and fixated using 4% (*w*/*v*) paraformaldehyde (PFA) at a pH of 7.2–7.4. After two final washing steps with PBS (10 min each), the stained tissue sections were covered with glycerol and a cover slip and stored at 4 °C in darkness till the images were captured.

### 2.5. Immunohistochemistry (IHC) of Paraffin-Embedded Tissue Sections

After rehydration in xylene and a subsequent descending ethanol series (100%, 90%, 80%, 70%), tissue sections were incubated in dilution buffer (PBS with 0.2% (*v*/*v*) BSA and 0.1% (*w*/*v*) NaN_3_) with the primary antibodies (Table 1) overnight at 4 °C. For negative controls, primary antibodies were omitted. After overnight incubation at 4 °C in darkness and three consecutive washing steps with PBS (10 min each), immunoreactivity was visualised using the EnVision kit (DAKO, Hamburg, Germany) followed by the nickel glucose oxidase approach [29,30].

### 2.6. vSMC Extraction

For vSMC extraction from the tunica media, a two-step enzymatic digestion was performed using collagenase (Collagenase Type II, Sigma-Aldrich). Again, aortae were dissected from the heart to the bifurcation and immediately transferred into MEM 1× + Pen/Strep stored on ice. Preparation of the tunica media was performed in cold MEM 1× buffer + Pen/Strep under a binocular microscope. Surrounding fat was removed using sterile tweezers, branching vessels were cut, and aortic tissue was first digested with collagenase (10% (*w*/*v*) in MEM 1× + Pen/Strep) at 37 °C for 15 min. Digestion was stopped by transferring the tissue into fresh MEM 1× + Pen/Strep. The tissue was separated manually from the tunica adventitia and bisected longitudinally to remove the inner tunica intima. The aortic media was cut into small pieces using sterile scissors. For the extraction of the SMCs, these pieces were transferred into fresh MEM1× + Pen/Strep with collagenase (10% (*w*/*v*) in MEM 1× + Pen/Strep) and incubated for 30 min at 37 °C. Afterwards, digestion was stopped by dilution with approximately 5 mL prewarmed cell culture medium DMEM/F-12 (Gibco, Thermo Fisher Scientific) containing 10% (*v*/*v*) foetal bovine serum (FBS, by Gibco, Thermo Fisher Scientific) + Pen/Strep. The cell suspension was strained through a cell strainer (40 µM grid size by BD Biosciences, Bedford, MA, USA) under sterile conditions, and the flow-through, containing the singularised vSMCs, was centrifuged for 10 min at 1500× *g* at room temperature. The pellet was resuspended in 5 mL fresh pre-warmed DMEM/F-12 + FBS + Pen-Strep and transferred into 25 cm^2^ sterile plastic cell culture flasks (Thermo Fisher Scientific).

### 2.7. Primary vSMC Culture

vSMCs were cultured at 37 °C and 5% CO_2_ with medium changed every 2–3 days. Extraction passage (P) was cultured for 6–7 days, and cells were trypsinised (Trypsin-EDTA solution, Sigma-Aldrich) for 5 min at 37 °C after gently washing (Dulbecco’s PBS (DPBS) by Gibco, Thermo Fisher Scientific). VSMCs (P1 and P2, after extraction) were seeded as follows: 4–5 × 10^5^ cells per 25 cm^2^ flask for RNA extraction and follow-up qPCR, 2 × 10^4^ cells per well (8-Well Chamber slide, Thermo Fisher Scientific) for IF staining.

### 2.8. IF Staining of vSMCs

Cultured vSMCs (P1, after extraction) were fixated for 10 min with 4% (*w*/*v*) PFA and washed 3 × 10 min with PBS while gently shaking. PBS was also used for the storage of fixated cells at 4 °C in the dark until staining. Cells were permeabilised with 0.1% (*v*/*v*) Triton (Merck, Darmstadt, Germany) in PBS for 10 min. Indirect immunostaining was performed by a two-step approach. To avoid non-specific binding of the antibodies, permeabilised vSMCs were blocked for one hour at room temperature with 2% (*v*/*v*) normal goat serum in PBS. Primary antibodies (Table 1) in dilution buffer were applied overnight at 4 °C in darkness. For negative controls the primary antibodies were omitted. After washing twice for 10 min with PBS, the secondary antibodies (Table 2) carrying the fluorescent tags Cy3 (excitation peak at 555 nm, dark orange in the visible spectrum) and Alexa 488 (excitation peak at 488 nm, green in the visible spectrum) diluted in PBS were applied for one hour at room temperature in darkness. Nuclear staining was performed together with the secondary antibodies using DAPI at a dilution of 1:1250 in PBS. The slides were washed twice with PBS (10 min each step) and fixated with 4% PFA for 10 min at room temperature and two subsequent PBS washing steps before preservation in glycerol and covering with a cover slip. Slides were stored at 4 °C in the dark until image capture. Fluorescence was observed and captured using a fluorescence microscope Axioskop 2 plus with the Axiovision LE software (Carl Zeiss, Goettingen, Germany).

### 2.9. RNA Isolation

Total RNA from cultured rat vSMCs (P1, after extraction) was prepared using the RNeasy^®^ Mini Kit (Qiagen, Hilden, Germany) according to manufacturer’s protocols for RNA isolation from animal cells (spin technology) with on-column DNase (Invitrogen, Thermo Fisher Scientific) digestion. 

The same kit was used for RNA isolation from frozen rat aorta following the manufacturer’s instructions for RNA isolation from animal tissue with on-column DNase digestion. Approximately 15–20 mg of rat aortae were frozen in liquid nitrogen and pulverised using a hammer. RLT buffer with 1% (*v*/*v*) 2-Mercaptoethanol (Roth, Karlsruhe, Germany) was added and, together with a sterile metal bead, the sample was processed for 3 min at 300 Hz in a tissue lyser mixer mill MM400 (Retsch, Haan, Germany). The lysate was centrifuged for 5 min at 12,000 rpm, and the supernatant was further processed according to the manufacturer’s protocol. RNAs were eluted in 30 µL RNase-free water, and RNA concentration was determined using the NanoDrop 2000 spectrophotometer (Thermo Fisher Scientific). RNA samples were stored at −80 °C. 

### 2.10. cDNA Synthesis

In total, 2 µg of total RNA served as template for cDNA synthesis. SuperScript^TM^ II RT and Oligo(dT)_20_ primer (Invitrogen, Thermo Fisher Scientific) were applied according to the manufacturer’s instructions using the Mastercycler gradient 5331 (Eppendorf, Hamburg, Germany). Consistency of the RNA extraction and efficiency of the RT-reaction was verified via determination of cDNA concentration using the NanoDrop 2000 spectrophotometer. When the concentration of the samples differed drastically, the cDNAs were adjusted to the same concentration for the subsequent qPCR measurement.

### 2.11. Primer Search for Potential RGs

The selection of potential RG candidates was based on the literature research. We considered studies that were performed using the model animal rat and the method qPCR. If indicated, we used the primer sequences for the RGs as described in these publications. If only the name of the RG was given, we used Primer BLAST for the respective primer design.

For every gene (Table 3), we determined the absolute Ct values of intact aortic media and the corresponding cultured vSMCs of four randomly chosen samples of each male and female animals. To minimise the influence of pipetting inaccuracies, the expression of each sample was measured as triplets. The absolute value of the difference between the Ct values of intact tissue and the cultured vSMCs (|Ct intact media − Ct cultured vSMCs|) served as preliminary indicator for gene expression stability of the RGs.

### 2.12. qPCR Primers and Calculation of Primer Efficiency

Primer pairs for GOI spanning an exon/exon junction were selected using Primer Blast (https://www.ncbi.nlm.nih.gov/tools/primer-blast/; accessed on 21 August 2021) or the respective references as indicated. Custom DNA-Oligos (0.01 µM, salt-free, lyophilised, MALDI-TOF MS quality analysis) were purchased from Eurofins, Hamburg, Germany. Details of final primer selection are given as follows:

**Actb:**

Forward 5′-ATCTGATACGTCCTCTATCC-3′

Reverse 5′-GTGGACGGAGCAAGCTCCTA-3′

Amplicon size: 232 bp

(BLAST: NM_031144.3)

**Gapdh:**

Forward 5′-CTTGTGCAGTGCCAGCCTC-3′

Reverse 5′-ACCAGCTTCCCATTCTCAGC-3′

Amplicon size: 230 bp

(BLAST: NM_017008.4)

**snRNP U5A:**

Forward 5′-ACTCTGGTTTCTCTTCAGATCG-3′

Reverse 5′-CAGAGTTGTTCCTCTCCA-3′

Amplicon size: 73 bp [31]

**snRNP U2:**

Forward 5′-ATCTGATACGTCCTCTATCC-3′

Reverse 5′-GTGGACGGAGCAAGCTCCTA-3′

Amplicon size: 83 bp [31]

The efficiency (**E**) of each primer pair was calculated based on a cDNA dilution series with three steps (undiluted, 1:2 and 1:4). Every dilution sample was measured as triplet according to the qPCR steps (Table 3). The resulting Ct mean values of each dilution level were plotted against the concentration of the diluted sample (log10 scale) and used to create a descending slope (**m**) by linear regression.

Primer efficiency calculation:

E = 10^[−1/m]^ − 1 (1)

An **m** of exactly −3.32 is necessary to achieve an efficiency of 100%. In theory the matrix material is duplicated in each reaction cycle, and with it, the fluorescence signal when an efficiency of 100% is assumed. We only used primer sets that achieved an efficiency of 90–110%.

### 2.13. qPCR

qPCR was performed using the iCycler IQ PCR system and the SYBR-Green Master mix (both by Bio-Rad, Hercules, CA, USA) using the cycler steps listed in Table 3.

**Figure A1:**
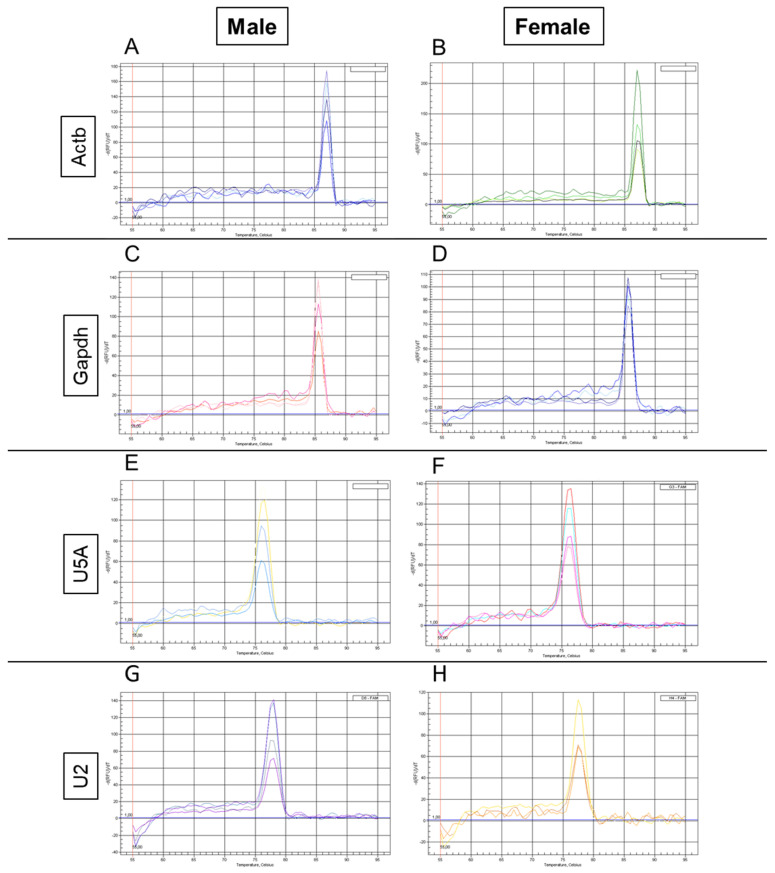

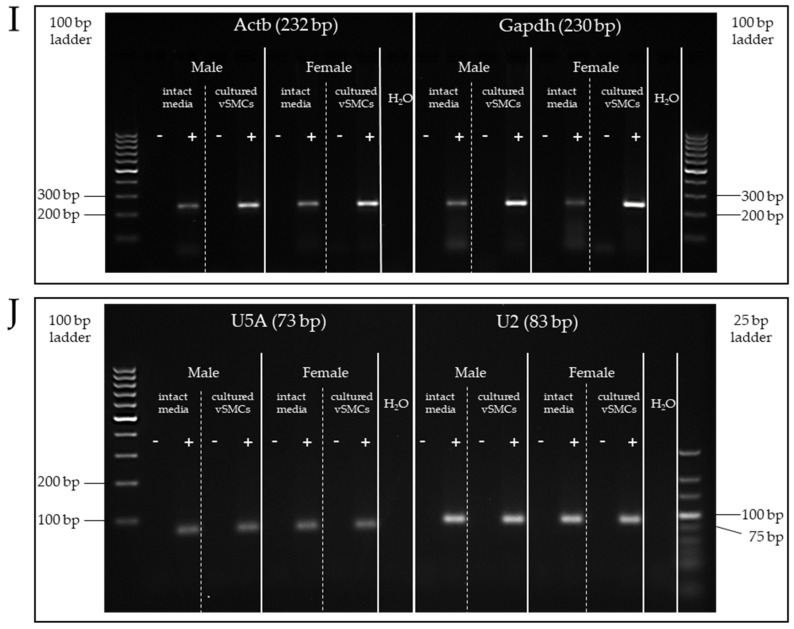
Melt curves of qPCR products (Actb, Gapdh, U5A, U2) of a randomly chosen exemplary male and female rat (**A**–**H**) and corresponding electrophoresis control gels (**I**,**J**) with (+) and without (–) RT. H2O symbolises the water control of the master mix for each primer set.

Electrophoresis using a 2% (*w*/*v*) agarose gel was performed to check for length of the qPCR products. Double stranded qPCR products were visualised with Ethidium bromide. Agarose gels as well as corresponding melt curves for Actb, Gapdh, U5A, and U2 are given in Figure A1

### 2.14. Statistics

For all underlying statistical analysis, GraphPad Prism 9 (Version 9.5.1 (733) for Windows, GraphPad Software, La Jolla, CA, USA, www.graphpad.com, accessed on 26 July 2023) was used. All the data were first checked for normal distribution. Detailed descriptions of statistical evaluation for each experiment are given in the corresponding figure legends.

Standard deviations from the common mean are indicated as real standard deviations (±SDs), not SEM. Differences were considered significant when (*) *p* < 0.05, (**) *p* < 0.01, (***) *p* < 0.001, and non-significant (ns) when *p* ≥ 0.05.

## 3. Results and Discussion

We aimed to realise the direct comparison between aortic vSMCs within intact tunica media and in culture at the level of transcription. Therefore, we tried to identify an RG candidate, which, due to its stable expression between these two groups, could serve as an internal control gene for normalisation when performing relative gene expression quantification and subsequent comparison between different samples. We extracted RNA from intact tunica media and cultured primary vSMCs of the same aortae from both male and female rats and compared the expression of various RG candidates (see Graphical Abstract).

### 3.1. Characterisation of Isolated and Cultured vSMCs

**Figure 1:**
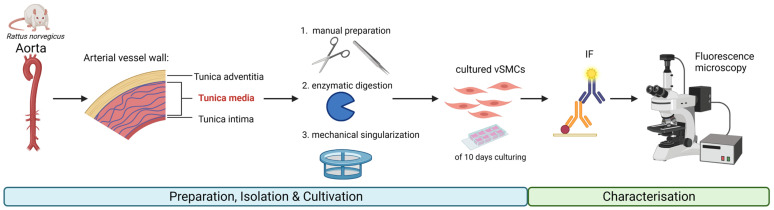
vSMC extraction from rat aortae, subsequent preparation for culture of primary vSMC, and characterisation by IF. Created with Biorender.

**Figure 2:**
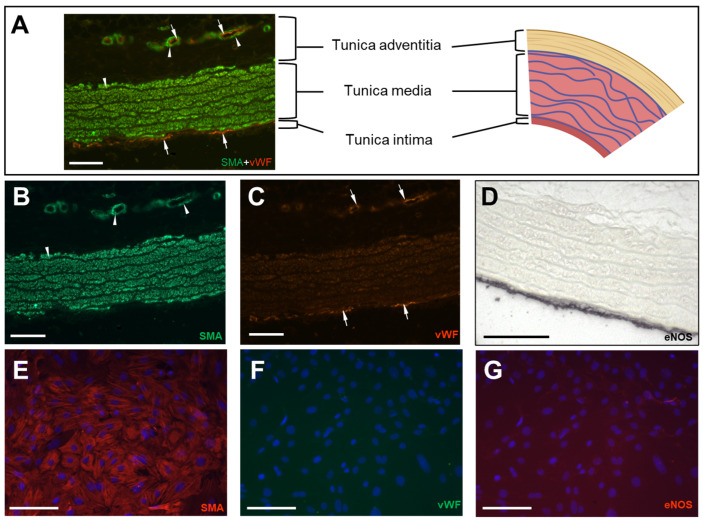
(**A**) Aortic cross section (schematic right, IF left with merged fluorescence channels for SMA (green) and vWF (red). (**B**) IF staining for SMA (arrowheads) and (**C**) orange staining for vWF (arrows)**.** Smaller blood vessels within the adventitia serve as internal positive control for endothelial (arrows) and vSMC staining (arrowheads). (**D**) IHC of the aorta for an additional endothelial marker (eNOS). (**E**–**G**) IF staining of cultured rat vSMCs of the first passage after extraction (10 days of culturing). IF staining of vSMCs for SMA ((**E**), red). No visible IF staining for endothelial markers vWF ((**F**), green) and eNOS ((**G**), red) confirms the absence of endothelial cells. Nuclei were stained blue with DAPI (**E**–**G**). Scale bar equals 100 µm. (**A**) Created with Biorender.

The aorta consists of an intima with its endothelial layer, a predominant media with vSMCs and extracellular elastic fibres, and an outer connective tissue layer, the adventitia (Figure 1 and Figure 2A).

**Figure A2:**
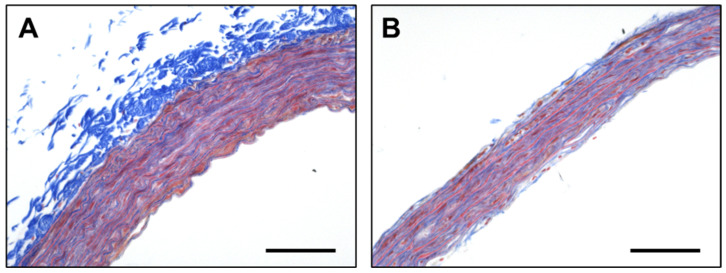
Azan staining of paraffin-embedded rat aorta cross sections (**A**) before and (**B**) after manual removal of the adventitia and intima following a 15 min treatment with collagenase. Scale bar equals 100 µm.

In efforts to isolate and culture exclusively vSMCs, we first removed the adventitia mechanically after incubation with collagenase for 15 min (Figure A2. The remaining vessel wall was opened longitudinally, and the intima was removed mechanically to obtain the tunica media of the vessel. After further extraction and cultivation steps, a homogenous culture of vSMCs (Figure 1) was confirmed by the absence of endothelial cells. This was verified by negative IF staining for vWF and eNOS (Figure 2F,G). In the intact vessel, however, vWF (Figure 2A,C) and eNOS (Figure 2D) were detectable in the inner layer. The smooth muscle identity of the cultured cells was confirmed by SMA (Figure 2E), which stained the media of the intact aorta as well (Figure 2B) [32,33].

### 3.2. Expression Levels of Routinely Used RGs Differ Significantly between Aortic vSMCs in Culture and Intact Media

**Figure 3:**
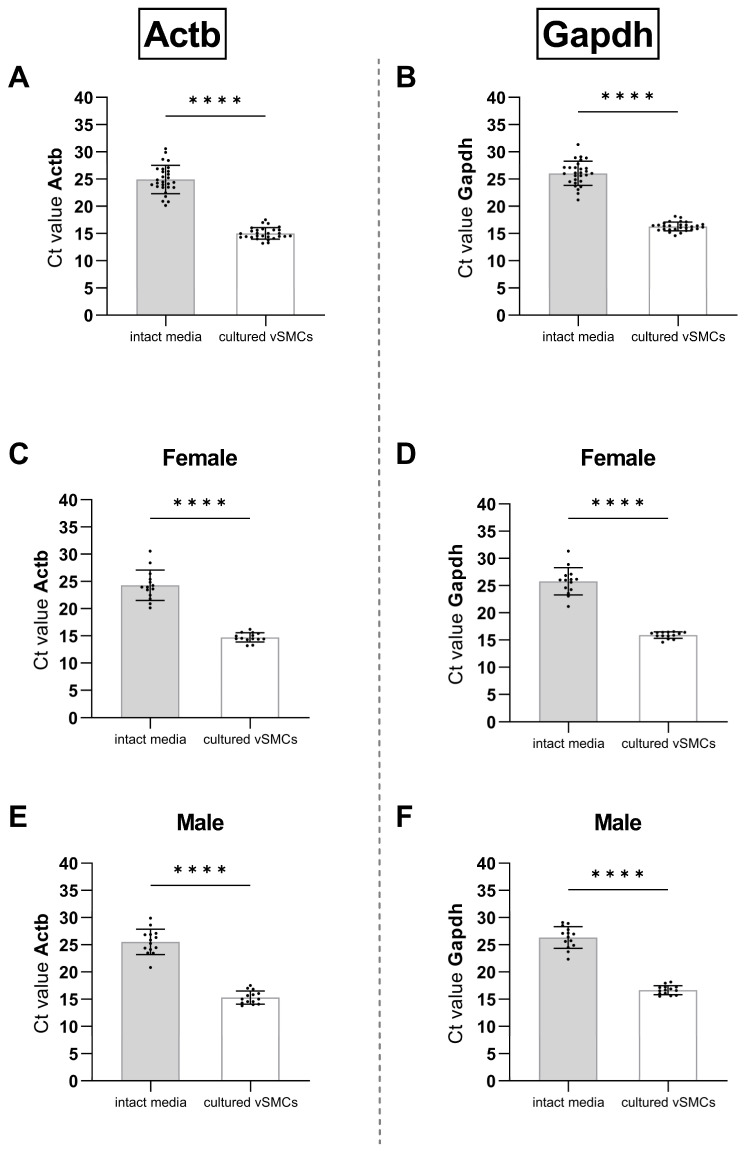
Comparison of the Ct values of the RGs Actb (left column) and Gapdh (right column) in intact aortic media (grey bars) and corresponding cultured vSMCs (white bars). Ct values of both, (**A**) Actb and (**B**) Gapdh were significantly higher (*p* < 0.0001, indicated by ****), in intact media compared to cultured vSMCs (paired two-tailed Student’s *t*-test; n = 28). The same result was found when comparing the expression of (**C**,**E**) Actb and (**D**,**F**) Gapdh in (**C**,**D**) female and (**E**,**F**) male rats separately (each n = 14). Standard deviations (±SD) are indicated b whiskers.

To perform a reliable comparison of gene expression between vSMCs in culture and tissue (intact media) an RG expressed stably between both groups is a prerequisite. We therefore initially tested whether the routinely used RGs Actb and Gapdh are expressed at a similar level and performed a pairwise comparison analysis (Figure 3).

The Ct values, inversely correlating to the level of gene expression, of both Actb (Figure 3A) and Gapdh (Figure 3B) in intact aortic media significantly exceeded the Ct values of the corresponding cultured vSMCs by approximately 10.

Since the template cDNA ideally is duplicated with each PCR cycle, the final amount of gene copies appears to be 2^10^ times higher in the cultured vSMCs for both Actb and Gapdh.

The rats analysed in Figure 3A,B consisted of both sexes. When analysing the females (Figure 3C,D) and males (Figure 3E,F) of this group separately, the difference in the Ct values of both Actb (Figure 3C,E) and Gapdh (Figure 3D,F) between intact tissue and the corresponding cultured cells remained significant and at a comparable range to the total group of animals (Figure 3A,B).

The observation of an increased RG expression in cultured cells was found to be independent of the sex. A potential difference would have required a conserved sex memory in cultured cells [34] since a steady exposure to the endocrine effects of the sex glands is missing.

For the comparison of gene expression between the samples of intact media, it should be carefully considered whether the large variance in expression in tissue allows the comparison of the respective GOIs. After all, the range of Ct values (~10) in intact tissue is almost as large as the difference between tissue and cells. Nevertheless, Actb, as well as Gapdh, may be suitable to compare gene expression either in the intact aortic media or vSMC culture.

The biological sex has not been considered as a relevant variable in research studies for too long. The concern that unpredictable hormonal changes in females (due to different cycle stages) may induce a broader variance of study results is a common argument of scientists not to involve females in their studies. However, researchers increasingly face the necessity to include male as well as female subjects in their studies to meet application criteria for funding. This seems justified since sex is an accepted risk factor for cardiovascular [35,36,37,38,39] and other diseases.

At the level of transcription, a sex bias might affect the expression of RGs [40] as well as GOIs. Therefore, studies targeting the expression of selected RGs (housekeeping genes) across different tissues and cells of males and females [40,41] are needed and constitute a fundamental contribution to basic research. To evaluate a potential sex dependency of the RG expression, our subsequent analyses were performed in male as well as female rats.

### 3.3. Identification of U2 as an RG Enabling a Direct Comparison of vSMCs in Intact Media and Culture

Despite an increasing number of publications on RGs (housekeeping genes) in rat tissue, hardly any studies comprise a direct comparison of cultured primary cells and their intact tissue of origin. Therefore, we searched for qPCR studies that compared rat tissue or cells before and after, for instance, drug administration and/or surgical intervention.

In total, 15 RGs (Table 4) of such studies were selected for an initial comparison of their expression in intact media and the corresponding cultured vSMCs. We are aware that databases, such as RefFinder [42], may provide an overview of promising RG candidates. Since our research question is rather specific and the Ct values recorded in such databases often only consider a tissue-specific expression, but not if these values were obtained in tissue and/or cultured cells, databases in our case may turn out to be of limited help. At the same time, this is also evidence that the direct comparison of gene expression of cultured cells and their tissue of origin is scarcely addressed by researchers.

We determined the Ct values of the selected genes (Table 4) in intact media and the corresponding cultured vSMCs. The absolute value of the difference between both Ct values (|Ct media − Ct vSMCs|) served as a preliminary indicator for the stability of the RG expression.

The average Ct difference between tissue and cells of all the genes tested was 4.5. Two-thirds of the RGs tested showed a difference of at least 4 Ct values. Since most of the genes are categorised as housekeeping genes, meaning essential to cell survival, it may indicate that the vSMCs undergo drastic physiological changes during extraction and cultivation, although the first phenotypical impression seems inconspicuous (Figure 2). This is what researchers studying vSMCs refer to as “phenotype switching” [20,22].

Nevertheless, according to our initial evaluation, the group of snRNPs was characterised by the most stable gene expression. It can therefore be assumed that snRNPs are of similar importance in vSMCs in culture and intact aortic media, which, as mentioned earlier, is a crucial prerequisite for our RG. The snRNPs are found in the splicing speckles and Cajal bodies of the eukaryotic nucleus, where they mainly contribute to the processing of pre-messenger RNAs [43,44]. The representative U5A and U2 showed the smallest Ct differences between tissue and cells (~0.5 Ct difference) both in male and female rats (Table 4).

**Figure 4:**
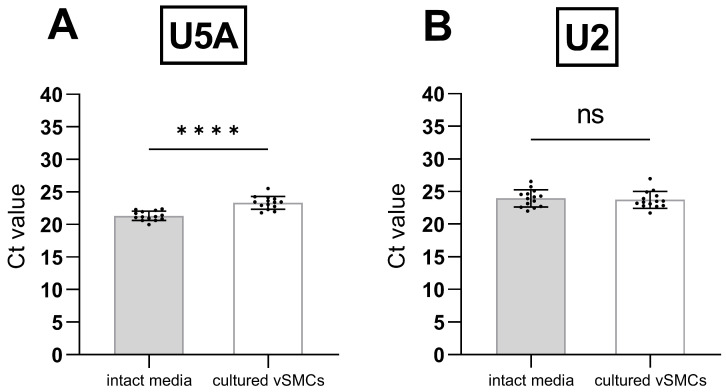
Comparison of the absolute Ct values of (**A**) U5A and (**B**) U2 in intact aortic media (grey bars, n = 14) and the corresponding cultured rat aortic vSMCs (white bars, n = 14). The comparison was performed by paired two-tailed Student’s *t*-test. Differences were considered significant when *p* < 0.0001, indicated by **** and non-significant (ns) when *p* > 0.05. Standard deviations (±SD) are indicated by whiskers.

To confirm these observations, we determined the absolute Ct values of U2 and U5A in a larger group of rats (Figure 4A,B; n = 14).

In contrast to the initial results (Table 4), the direct comparison by paired Student’s *t*-test revealed significant differences between the Ct values of U5A in aortic tissue versus cells (Figure 4A). The average difference was ~2 Ct values. Thus, unfortunately, U5A also proved to be non-suitable as an RG for the comparison of tissue and cells.

For the second candidate U2, however, statistical analyses of a larger set of samples did not reveal a significant Ct difference between tissue and the corresponding cells (Figure 4B), thereby classifying U2 as suitable RG for the comparison of cultured versus tissue vSMCs. Additionally, this also shows that even in the same group of genes, expression differences should not be excluded categorically.

**Figure 5:**
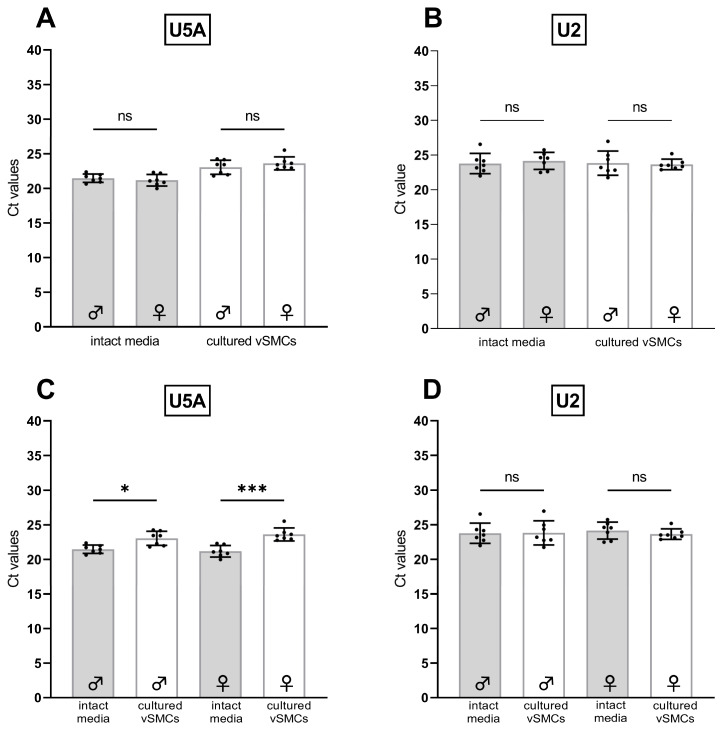
(**A**,**C**) Comparison of absolute Ct values of U5A and (**B**,**D**) U2 in the intact aortic media (grey bars) and the corresponding cultured vSMCs (white bars) of male (♂, n = 7) and female (♀, n = 7) rats (Tukey’s multiple comparisons test). Only relevant comparisons are displayed. Significant differences are indicated as *p* ≤ 0.05 *, *p* < 0.001 ***, or non-significant (ns). Standard deviations (±SD) are indicated by whiskers.

When comparing the expression of U5A (Figure 5A) and U2 (Figure 5B) of samples from intact tissue or of samples from cultured vSMCs, while taking the sex into account, no significant difference neither in intact tissue nor cultured vSMCs was found between male and female rats (n = 7, each). This suggests that U5A and U2 are eligible as RGs for the comparison between male and female samples of either intact media or of cultured vSMCs.

When comparing male and female rats regarding the expression of U5A (Figure 5C) and U2 (Figure 5D) in samples from intact tissue and the corresponding cultured vSMCs, U5A was expressed significantly higher in the intact tissue of both sexes. On the other hand, U2 showed no significant difference in its expression neither in males nor females.

## 4. Conclusions and Outlook

The changes in all RGs we tested (Table 4) indicate that most routinely used RGs are unsuitable for the direct comparison of vSMCs in intact tissue versus culture. Furthermore, these discrepancies might reflect phenotype switching of vSMCs in the context of culturing. While cell culture remains an important part of basic research, the selection of RGs used for in vitro experiments should be validated consciously. We now could identify U2 as a suitable RG that allows the comparison between intact aortic media and cultured vSMCs regardless of the sex of the study subjects.

A relevant task besides the detailed evaluation of RGs is the improvement of tools that support the selection of appropriate RGs for different experimental settings. It would be of utmost importance to continue updating tools like RefFinder [42] with recently validated data to generate a constantly evolving database that grows in detail and precision over time.

## Figures and Tables

**Table 1:** Primary antibodies for immunostainings.

Antibody	Host Antigen	Cat.no.	Clonality	Manufacturer	Dilution for Cells	Dilution for Tissue
anti-Calponin	rabbit	ab46794	Mono	Abcam, Cambridge, UK	1:500	1:500
anti-SMA	mouse	A5228	Mono	Sigma-Aldrich, St. Louis, MO, USA	1:1000	1:1000
anti-vWF	rabbit	AB7356	Poly	Sigma-Aldrich, St. Louis, MO, USA	1:500	1:1000
anti-eNOS	mouse	610297	Mono	BDTrans, Franklin Lakes, NJ, USA	1:500	1:200

Endothelial nitric oxide synthases (eNOS), smooth muscle actin (SMA), von Willebrand factor (vWF).

**Table 2:** Secondary antibodies for immunostainings.

Antibody	Host Antigen	Cat.no.	Clonality	Manufacturer	Dilution for Cells	Dilution for Tissue
anti-rabbit Alexa488	goat	A-11034	Poly	Invitrogen, Waltham, MA, USA	1:250	1:500
anti-rabbit Cy3		111-166-045	Poly	Jackson Immuno Research, Cambridgeshire, UK		
anti-mouse Alexa488		A-11001	Poly	Invitrogen		
anti-mouse Cy3		115-165-003	Poly	Jackson Immuno Research		

**Table 3:** Cycler steps for qPCR.

	Step	Cycles	Duration	Temperature
1	Polymerase activation	1×	3 min	95 °C
2	Denaturation	35×	20 s	95 °C
3	Primer hybridisation		20 s	60 °C
4	Elongation		20 s	72 °C
5	Melt curve analysis			55–95 °C

**Table 4:** This table provides relevant information about the 15 selected genes and their primer sets tested as potential RGs. The absolute values of the difference of Ct values (intact aortic media - corresponding cultured vSMCs) served as an indicator for the stability of their expression. In total 8 animals were analysed (male and female, each n = 4). The heat scale ranks the difference values from high (dark red) to low (dark green).

Gene	Gene Group	Primer	|Ct _intact media_ − Ct _cultured vSMCs_|		
			Total	Male	Female
Hypoxanthine phosphoribosyltransferase 1	nucleotide synthesis	Hprt1_4	6.45	4.51	8.40
		Hprt1_B	5.16	4.48	5.84
Nucleoporin like 2	protein export from nucleus	Nupl2_B	6.29	7.48	5.48
Tankyrase	protein stability and interaction	Tnks_T	7.42	7.30	7.55
		Tnks_1	6.37	5.81	6.92
TATA box binding protein	transcription	Tbp_T	6.19	6.17	6.20
Ribosomal protein L30	protein synthesis	Rpl30_3	6.51	6.35	6.67
		Rpl30_1	5.55	5.78	5.83
Ribosomal protein L32		Rpl32_4	4.77	4.69	4.84
		Rpl32_1	3.78	2.56	5.00
Actin alpha 2, smooth muscle	cell structure and motility	Acta2_2	4.53	4.43	4.64
		Acta2_3	3.66	2.90	4.41
Beta-2-microglobulin	serum protein	B2m_9	4.28	4.19	4.37
		B2m_T	3.75	3.40	4.10
Eukaryotic translation elongation factor 1 alpha 1	translation	Eef1a1_T	4.25	4.08	4.42
Eukaryotic translation elongation factor 2		Eef2_1	4.60	4.04	5.16
		Eef2_2	4.58	4.39	4.76
DNA topoisomerase 1	DNA topology during transcription	Top1_B	5.42	5.45	5.79
		Top1_1	3.66	3.49	3.84
Small nucleolar RNA, C/D box 87	methylation of preribosomal RNA precursors	Snord87_T	2.92	3.22	2.66
5S ribosomal RNA	ribosomal RNAs	5SrRNA_T	2.37	2.07	2.33
Small nuclear RNA U5A	splicing	U5A_T	0.99	0.58	1.11
Small nuclear RNA U2		U2_T	0.31	0.39	0.23
